# Progress Toward Rubella and Congenital Rubella Syndrome Control — South-East Asia Region, 2000–2016

**DOI:** 10.15585/mmwr.mm6721a3

**Published:** 2018-06-01

**Authors:** Sudhir Khanal, Sunil Bahl, Mohammad Sharifuzzaman, Deepak Dhongde, Sirima Pattamadilok, Susan Reef, Michelle Morales, Alya Dabbagh, Katrina Kretsinger, Minal Patel

**Affiliations:** ^1^Expanded Programme on Immunization, World Health Organization South-East Asia Regional Office, Delhi, India; ^2^Global Immunization Division, Center for Global Health, CDC; ^3^World Health Organization, Geneva, Switzerland.

In 2013, the 66th session of the Regional Committee of the World Health Organization (WHO) South-East Asia Region (SEAR)* adopted the goal of elimination of measles and control^†^ of rubella and congenital rubella syndrome (CRS) by 2020 (*1*). Rubella is the leading vaccine-preventable cause of birth defects. Although rubella typically causes a mild fever and rash in children and adults, rubella virus infection during pregnancy, especially during the first trimester, can result in miscarriage, fetal death, or a constellation of congenital malformations known as CRS, commonly including visual, auditory, and/or cardiac defects, and developmental delay (*2*). Rubella and CRS control capitalizes on the momentum created by pursuing measles elimination because the efforts are programmatically linked. Rubella-containing vaccine (RCV) is administered as a combined measles and rubella vaccine, and rubella cases are detected through case-based surveillance for measles or fever and rash illness (*3*). This report summarizes progress toward rubella and CRS control in SEAR during 2000–2016. Estimated coverage with a first RCV dose (RCV1) increased from 3% of the birth cohort in 2000 to 15% in 2016 because of RCV introduction in six countries. RCV1 coverage is expected to increase rapidly with the phased introduction of RCV in India and Indonesia beginning in 2017; these countries are home to 83% of the SEAR birth cohort. During 2000–2016, approximately 83 million persons were vaccinated through 13 supplemental immunization activities (SIAs) conducted in eight countries. During 2010–2016, reported rubella incidence decreased by 37%, from 8.6 to 5.4 cases per 1 million population, and four countries (Bangladesh, Maldives, Sri Lanka, and Thailand) reported a decrease in incidence of ≥95% since 2010. To achieve rubella and CRS control in SEAR, sustained investment to increase routine RCV coverage, periodic high-quality SIAs to close immunity gaps, and strengthened rubella and CRS surveillance are needed.

## Immunization Activities

Before 2000, only two of the 11 SEAR countries (Sri Lanka and Thailand) included RCV in the routine infant immunization schedule. By the end of 2016, eight (73%) countries had introduced RCV (Table 1). India, Indonesia, and North Korea, three countries that include 84% of infants living in the region, had not yet introduced RCV, but India and Indonesia plan to introduce RCV in the immunization schedule in phases during 2017–2019. The age of administration is at age 9–9.5 months for RCV1 and 15–36 months for the second RCV dose (Table 1). WHO and the United Nations Children’s Fund (UNICEF) use reported administrative coverage of RCV1 (i.e., the number of doses administered divided by the estimated target population) along with survey data to estimate national RCV1 coverage (*4*,*5*). Estimated regional RCV1 coverage of the birth cohort in the region increased from 3% in 2000 to 15% in 2016 (Figure). Six of eight countries that had introduced RCV1 by 2016 reported ≥90% coverage nationwide (Table 1). During 2000–2016, eight SEAR countries conducted SIAs and vaccinated 83.1 million children, adolescents, and young adults (Table 2).

**TABLE 1:** Estimated coverage* with rubella-containing vaccine (RCV), age at vaccination, number of confirmed rubella and congenital rubella syndrome (CRS) cases, and rubella incidence, by country — World Health Organization South-East Asia Region, 2010 and 2016

Country (year RCV introduced)	2010					2016					% change in rubella incidence 2010 to 2016
	% RCV1 coverage	RCV schedule	No. of confirmed CRS cases	No. of confirmed rubella cases	Rubella incidence^†^	% RCV1 coverage	RCV schedule	No. of confirmed CRS cases	No. of confirmed rubella cases	Rubella incidence^†^	
Bangladesh (2012)	NA^§^	NA	NR^¶^	12,963	87.4	94	9.5m, 15m	87	165	1.0	-99
Bhutan (2006)	95	9m, 24m	NR	9	12.9	97	9m, 24m	0	3	4.0	-69
India (N/A)	NA	NA	NR	NR	NR	NA	NA	25	8,274	6.4	—
Indonesia (N/A)	NA	NA	NR	1,323	5.6	NA	NA	174	1,238	4.8	-15
Maldives (2007)	96	9m, 18m	NR	4	12.5	99	18m	0	0	0.0	-100
Myanmar (2015)	NA	NA	NR	11	0.2	91	9m	0	10	0.2	0
Nepal (2013)	NA	NA	NR	510	18.5	83	9m, 15m	33	656	22.9	+24
North Korea (N/A)	NA	NA	NR	0	0.0	NA	NA	0	0	0.0	0
Sri Lanka (1996)	99	3y, 13y	8	68	3.3	99	9m, 3y	0	0	0.0	-100
Thailand (1993)	98	9m, p1	NR	387	6.1	99	9m, 2.5y	0	7	0.1	-98
Timor-Leste (2016)	NA	NA	NR	NR	NR	78	9m, 18m	0	8	6.5	—
**South-East Asia Region**	**3**	**—**	**8**	**15,275**	**8.6**	**15**	**—**	**319**	**10,361**	**5.4**	**-37**

**Source:** http://www.who.int/immunization/monitoring_surveillance/data/en.

**Abbreviations:** m = months; NA = not applicable; NR = not reported; p = primary grade of school; RCV1 = first dose of RCV; y = years.

* Data are from World Health Organization and United Nations Children’s Fund (UNICEF) estimates, 2016 revision (as of July 2017).

^†^ Cases per 1 million population.

^§^ Dose was not included in the vaccination schedule for that year.

^¶^ Country did not report cases in the year specified.

**FIGURE:**
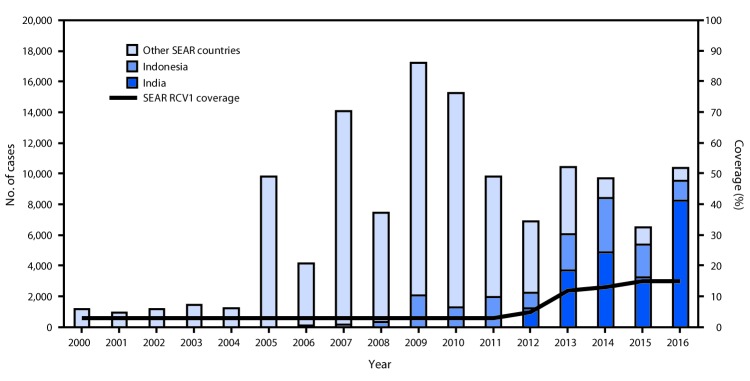
Number of reported rubella cases,* by country, and estimated first dose rubella-containing vaccine (RCV1)^†^ coverage — World Health Organization (WHO) South-East Asia Region (SEAR),^§^ 2000–2016 **Source:** http://www.who.int/immunization/monitoring_surveillance/data/en. **Abbreviation:** RCV = rubella-containing vaccine in routine immunization. * Cases of rubella reported to WHO and the United Nations Children’s Fund (UNICEF) through the Joint Reporting Form to the Regional Office for the South-East Asia Region. ^†^ Data are from WHO and UNICEF estimates for SEAR. ^§^ Other countries in the region include Bangladesh, Bhutan, Maldives, Myanmar, Nepal, North Korea, Sri Lanka, Thailand, and Timor-Leste.

**TABLE 2:** Characteristics of rubella supplementary immunization activities (SIAs),* by country and year — World Health Organization (WHO) South-East Asia Region, 2000–2016

Country	Year	Rubella-containing vaccine used	SIA type	SIA extent	Target age group	Population reached in targeted age group	% administrative coverage
Bangladesh	2014	MR	Catch-up	National	9m–15y	53,644,603	>100^†^
	2016	MR	Follow-up	Subnational	9m–5y	100,863	>100^†^
Bhutan	2006	MR	Catch-up	National	9m–14y; 15y–44y F	332,041	98
Maldives	2005	MR	Catch-up	National	6y–25y M; 6y–35y F	118,877	82
	2006	MR	Catch-up	National	6y–25y M; 6y–35y F	123,642	85
	2007	MMR	Follow-up	National	4y–6y	16,462	56
Myanmar	2015	MR	Catch-up	National	9m–15y	13,160,764	94
Nepal	2012	MR	Catch-up	National	9m–15y	8,524,991	89
	2015	MR	Follow-up	Subnational	6m–15y	453,665	91
	2016	MR	Follow-up	Subnational	9m–5y	2,528,539	>100^†^
Sri Lanka	2004	MR	Catch-up	National	16y–20y	1,362,108	72
Thailand	2015	MR	Follow-up	National	2.5y–7y	2,244,906	88
Timor-Leste	2015	MR	Catch-up	National	6m–15y	484,850	97
**South-East Asia Region**						**83,096,311**	**98**

**Source:** http://www.who.int/immunization/monitoring_surveillance/data/en.

**Abbreviations:** F = females; M = males; MMR = measles, mumps, and rubella vaccine; MR = measles and rubella vaccine; m = months; y = years.

* Rubella SIAs generally are carried out along with measles SIAs using two target age ranges. An initial, nationwide catch-up SIA targets all children aged 9 months–15 years, with the goal of eliminating susceptibility to rubella virus in the general population. Periodic follow-up SIAs then target all children born since the last SIA. Follow-up SIAs generally are conducted nationwide every 2–4 years and target children aged 9–59 months; their goal is to eliminate any rubella virus susceptibility that has developed in recent birth cohorts and to protect children who did not respond to the first rubella vaccination.

^†^ Values >100% indicate that the intervention reached more persons than the estimated target population. The numerator was the total children vaccinated, and the denominator was the estimated target calculated for vaccination.

## Surveillance Activities

Rubella cases and outbreaks were reported by three countries (Bhutan, Sri Lanka, and Thailand) in 2000, by nine countries (all but India and Timor-Leste) in 2010, and by all 11 countries in 2013. By 2016, case-based measles-rubella surveillance had been initiated in all SEAR countries and included rubella immunoglobulin M (IgM) antibody testing for all suspected measles cases^§^ that tested negative for measles IgM antibody. Countries reported measles-rubella case-based surveillance data indicators^¶^ to the WHO SEAR office (*6*,*7*). A SEAR measles-rubella laboratory network with eight participating laboratories was established in 2003 as part of the WHO Global Measles and Rubella Laboratory Network. By 2016, the network had expanded to include one regional reference laboratory in Thailand and 39 proficient** national or subnational laboratories (13 in India, four in Indonesia, 14 in Thailand, and one in each of the other eight countries).

The number of SEAR countries reporting CRS cases through the WHO-UNICEF Joint Reporting Form (JRF)^††^ increased from two in 2002 to 10 in 2016. North Korea, Sri Lanka, and Thailand report CRS cases as part of the national integrated disease surveillance programs. Eight countries identify CRS cases through sentinel site surveillance (Bangladesh, since 2012; Indonesia and Nepal, 2014; Maldives, 2015; Bhutan, India, Myanmar and Timor-Leste, 2016). Bangladesh also has population-based CRS surveillance, for which all vaccine-preventable disease surveillance reporting sites also report CRS cases.

## Rubella Incidence and Rubella Virus Genotypes

From 2010 to 2016, reported annual rubella incidence in SEAR decreased 37%, from 8.6 to 5.4 cases per 1 million population. Five countries reported <1 rubella case per 1 million population in 2016, including four (Bangladesh, Maldives, Sri Lanka, and Thailand) that reported a decrease in incidence of ≥95% since 2010 (Table 1). In 2016, SEAR countries reported 10,361 laboratory confirmed and epidemiologically linked rubella cases, including 1,720 sporadic cases and 8,641 cases that occurred in 263 laboratory-confirmed rubella outbreaks and 68 mixed measles and rubella outbreaks. Only five of the 8,641 confirmed outbreak-associated rubella cases occurred in countries that had introduced RCV. Among the confirmed outbreak-associated cases, 698 (8%) patients were aged <1 year; 2,682 (31%), 1–4 years; 3,297 (38%), 5–9 years; 1,207 (14%), 10–14 years; and 757 (9%), ≥15 years. Overall, 7,884 (91%) of the outbreak-associated cases in 2016 occurred in children aged <15 years. Among all reported rubella cases in 2016, a total of 9,512 (92%) occurred in India and Indonesia (Figure). Reported CRS cases increased from 26 in 2002 to 319 in 2016, reflecting an increase in countries reporting CRS cases from two in 2002 to 10 in 2016 (Table 1). During 2000–2016, 84 rubella viruses (all genotypes 1E or 2B) were reported from the region to the Rubella Nucleotide Sequence Database (RubeNS).^§§^

## Discussion

Substantial progress was made toward rubella and CRS control in SEAR during 2000–2016, with a 37% decline in reported rubella incidence. Momentum for rubella and CRS control was accelerated by the Regional Committee with the establishment of a regional goal in 2013 to achieve measles elimination and rubella and CRS control by 2020 (*1*). After this goal was established, countries rapidly introduced RCV, and eight of 11 countries now include RCV in the routine immunization schedule. In four countries (Bangladesh, Maldives, Sri Lanka, and Thailand) rubella and CRS likely have been controlled. Fifteen percent of the SEAR birth cohort received RCV through routine immunization services in 2016; with the introduction of RCV in India and Indonesia beginning in 2017, regional RCV1 coverage is expected to increase rapidly.

In the SEAR countries, rubella cases occurred mostly among children aged <15 years; catch-up SIAs conducted in Bangladesh, Bhutan, Myanmar, Nepal, and Timor-Leste during 2000–2016 targeted this age group and achieved overall decreases in rubella incidence. Therefore, rubella incidence is expected to decrease significantly when the populous countries of India and Indonesia conduct catch-up SIAs as part of RCV introduction into the immunization programs. Periodic high-quality SIAs will be needed to close immunity gaps until high measles and rubella vaccination coverage is achieved through routine immunization services by all countries in the region. Sustained investments to achieve or maintain high routine RCV coverage are needed.

Optimal surveillance for rubella and CRS is essential to monitor the impact of rubella vaccine introduction to ensure that there is no epidemiologic age shift in incidence (from children to women of childbearing age) and to verify progress toward rubella and CRS control goals. As countries progress toward elimination of endemic rubella virus transmission, elimination-standard surveillance will be required (*8*). Efforts needed to achieve this include modifying the case definition to include all cases of rash and fever from both public and private sector clinical sites and enhancing laboratory capacity to support surveillance, including the ability to process an increased number of specimens following the change to a more sensitive case definition.

The findings in this report are subject to at least two limitations. First, 30%–50% of rubella virus infections are typically asymptomatic or mild; thus many rubella cases are likely not to be detected and reported (*2*). CRS surveillance complements rubella surveillance data and improves monitoring of rubella disease burden in the population. Second, the quality of surveillance varies among countries, and the definition used for suspected rubella cases varies from country to country, which limits comparisons of surveillance data among countries. 

The midterm review of the Strategic Plan for Measles Elimination and Rubella/CRS Control for WHO South-East Asia Region 2014–2020 found evidence that four countries (Bangladesh, Maldives, Sri Lanka, and Thailand) had achieved ≥95% reduction in rubella cases since 2010 (*9*). The regional goal of rubella and CRS control by 2020 appears to be achievable; with continued investment in high routine RCV coverage, periodic high-quality SIAs, and improved rubella and CRS surveillance, a regional rubella elimination goal might be considered in the near future.

**Summary**

**What is already known about this topic?**

Before 2000, only two World Health Organization South-East Asia Regional (SEAR) countries had introduced rubella-containing vaccine (RCV) into routine immunization programs.

**What is added by this report?**

During 2000–2016, six additional SEAR countries introduced RCV, and first dose RCV (RCV1) coverage increased from 3% (2000) to 15% (2016). During 2010–2016, reported rubella incidence decreased 37%, from 8.6 to 5.4 cases per 1 million population. Bangladesh, Maldives, Sri Lanka, and Thailand likely have controlled rubella and congenital rubella syndrome (CRS).

**What are the implications for public health practice?**

Rubella and CRS elimination in the region might be considered with investment in high routine RCV coverage, periodic high-quality supplementary immunization activities, and improved rubella and CRS surveillance. With the introduction of RCV in India and Indonesia beginning in 2017, regional RCV1 coverage is expected to increase rapidly.

## Footnotes

* The WHO South-East Asia Region consists of 11 countries: Bangladesh, Bhutan, India, Indonesia, Maldives, Myanmar, Nepal, North Korea, Sri Lanka, Thailand, and Timor-Leste.

† Rubella and CRS control is defined as a 95% reduction of rubella and CRS cases compared with the 2010 baseline nationally and regionally.

§ A suspected measles case was defined as any case of fever and maculopapular rash in Bhutan, Maldives, Myanmar, and Timor-Leste. The remaining countries used a suspected measles case definition that included fever, maculopapular rash, and any of the following: cough, coryza, or conjunctivitis.

¶ Surveillance indicators include 1) an annualized incidence rate of two discarded nonmeasles nonrubella cases per 100,000 population at the national level; 2) ≥80% of subnational administrative units reporting ≥2 discarded nonmeasles nonrubella cases per 100,000 population per year; 3) ≥80% of suspected cases tested for measles and rubella IgM antibodies; 4) ≥80% of laboratory-confirmed chains of transmission have adequate samples collected for detecting rubella virus and tested in an accredited laboratory; and 5) ≥80% of suspected cases have an adequate investigation conducted within 48 hours of notification.

** A proficient laboratory is one that has been defined as having met defined criteria as outlined in the report, “Framework for verifying elimination of measles and rubella.” http://www.who.int/wer/2013/wer8809.pdf.

†† The JRF is used yearly by countries to report information on immunization schedules, vaccination campaigns, number of vaccine doses administered through routine immunization services, and other monitoring data to WHO and UNICEF. Surveillance data, including number of cases of rubella and CRS, are also reported to WHO and UNICEF through the JRF using standard case definitions.

§§ RubeNS and the Measles Nucleotide Surveillance Database (MeaNS) are components of laboratory surveillance for measles and rubella; all laboratories in the Global Measles Rubella Laboratory Network are requested to submit nucleotide sequences for viruses identified.
